# Registered nurses’ application of the principles of evidence-based practice the first five years after graduation

**DOI:** 10.1186/1472-6963-14-S2-P36

**Published:** 2014-07-07

**Authors:** Anna Ehrenberg, Lars Wallin, Petter Gustavsson, Ann Rudman, Anne-Marie Boström

**Affiliations:** 1 School of Education, Health and Social Studies, Dalarna University, Falun, Sweden; 2 Department of Clinical Neuroscience, Karolinska Institutet, Stockholm, Sweden; 3 Department of Neurobiology, Care Science and Society, Division of Nursing, Karolinska Institutet, Stockholm, Sweden; 4 Danderyd Hospital, Department of Geriatric Medicine, Danderyd, Sweden

## Background

It has been proposed that the capacity to provide evidence-based practice is one of five core competencies that all healthcare professions should possess to meet the needs of the 21st century healthcare system. New nurses are faced with a challenging work environment which, combined with shortcomings in undergraduate education and their limited clinical experience, may affect their evidence-based practice. The aim of this study was to prospectively examine the extent of Swedish nurses’ evidence-based practice during the first five years of professional life.

## Materials and methods

This was an observational longitudinal study, with yearly data collections over the course of five years. Data was collected in two national cohorts (named EX2004 and EX2006) of Swedish registered nurses. They had completed a three year academic nursing program and mainly worked in hospital settings. Participants were recruited while studying at any of the 26 universities in Sweden. A total of 2107 (EX2006) and 2331 (EX2004) nursing students were eligible. 1207 and 1227 nurses were included in the current longitudinal samples. The nurses had a mean age of 31.2/33.9 years old and a majority were female. The cohorts were representative of the general nursing population. Data was self-reported and collected through annual postal surveys. Evidence-based practice was conceptualized as a process and measured with an instrument including six items. Data was analyzed using latent growth curve modelling.

## Results

Implementation of evidence-based practice was stable, between the two cohorts and over time. Individual differences existed and remained stable over time. However, the extent of practicing the different components of evidence-based practice on a monthly basis varied considerably, from 10% of the nurses (appraising research reports) to 80% (using information sources other than databases to search for knowledge).

## Conclusion

The extent of evidence-based practice remained unchanged during the first five years of professional life. It appears important to enhance both the contribution of undergraduate education and the contextual conditions in work life, in order to improve evidence-based practice among newly graduated nurses.

